# Cyclic GMP-AMP synthase promotes the inflammatory and autophagy responses in Huntington disease

**DOI:** 10.1073/pnas.2002144117

**Published:** 2020-06-24

**Authors:** Manish Sharma, Sumitha Rajendrarao, Neelam Shahani, Uri Nimrod Ramírez-Jarquín, Srinivasa Subramaniam

**Affiliations:** ^a^Department of Neuroscience, The Scripps Research Institute, Jupiter, FL 33458

**Keywords:** striatal vulnerability, *HTT*-copy number, cotranslational cleavage, autophagy flux, pro-inflammatory response

## Abstract

Huntington disease (HD) is caused by an expansion mutation of the N-terminal polyglutamine of huntingtin (mHTT). mHTT is ubiquitously present, but it induces noticeable damage to the brain’s striatum, thereby affecting motor, psychiatric, and cognitive functions. The striatal damage and progression of HD are associated with the inflammatory response; however, the underlying molecular mechanisms remain unclear. Here, we report that cGMP-AMP synthase (cGAS), a DNA sensor, is a critical regulator of inflammatory and autophagy responses in HD. Ribosome profiling revealed that the *cGAS* mRNA has high ribosome occupancy at exon 1 and codon-specific pauses at positions 171 (CCG) and 172 (CGT) in HD striatal cells. Moreover, the protein levels and activity of cGAS (based on the phosphorylated STING and phosphorylated TBK1 levels), and the expression and ribosome occupancy of cGAS-dependent inflammatory genes (*Ccl5* and *Cxcl10*) are increased in HD striatum. Depletion of cGAS diminishes cGAS activity and decreases the expression of inflammatory genes while suppressing the up-regulation of autophagy in HD cells. In contrast, reinstating cGAS in cGAS-depleted HD cells activates cGAS activity and promotes inflammatory and autophagy responses. Ribosome profiling also revealed that *LC3A* and *LC3B*, the two major autophagy initiators, show altered ribosome occupancy in HD cells. We also detected the presence of numerous micronuclei, which are known to induce cGAS, in the cytoplasm of neurons derived from human HD embryonic stem cells. Collectively, our results indicate that cGAS is up-regulated in HD and mediates inflammatory and autophagy responses. Thus, targeting the cGAS pathway may offer therapeutic benefits in HD.

## Significance

Huntington disease (HD) is a genetic disorder caused by glutamine-expansion in the huntingtin (mHTT) protein, which affects motor, psychiatric, and cognitive function, but the mechanisms remain unclear. mHTT is known to induce DNA damage and affect autophagy, both associated with inflammatory responses, but what mediates all these were unknown. Here we report that cGAS, a DNA damage sensor, is highly upregulated in the striatum of a mouse model and HD human patient’s tissue. We found ribosomes, which make proteins, are robustly accumulated on the cGAS mRNA in HD cells. cGAS depletion decreases—and cGAS expression increases—both inflammatory and autophagy responses in HD striatal cells. Thus, cGAS is a therapeutic target for HD. Blocking cGAS will prevent/slow down HD symptoms.

Huntington disease (HD) is a fatal neurodegenerative disorder that is caused by CAG expansion mutation of the Huntingtin gene (*HTT*), which codes for polyglutamine. The mutant gene, *mHTT*, is ubiquitously expressed, but it causes prominent damage to the striatum and cortex and subsequently leads to widespread peripheral defects as the disease progresses. Inflammatory responses have been implicated in the pathogenesis of HD (1–6), and compounds with anti-inflammatory properties have been shown to improve survival in HD transgenic mice (7, 8). The number of microglia, a cellular indicator of inflammation, has been shown to be increased in the striatum of HD animal models, cell culture models, and HD patients (9–11). Additionally, the levels of reactive monocytes, inflammatory cytokines, chemokines, and the n-kynurenine/tryptophan ratio, which is an indicator of persistent inflammation, are all increased in premanifest HD patients, and are correlated with HD progression (12–15). Studies in mice indicate that glial cells and galectin molecules contribute to enhanced inflammatory responses in HD (16, 17). Furthermore, RNA-sequencing (RNA-seq) analysis of tissue obtained from human HD patients and HD monkeys revealed extensive transcriptional dysregulation associated with the activation of pro-inflammatory pathways (1, 18, 19). Inflammation is also closely linked to autophagy, a catabolic process that is dysregulated in HD (20, 21). These findings indicate that inflammation is a prominent cellular response in HD patients and across various HD models, although the mechanisms are not entirely clear.

cGMP-AMP synthase (cGAS, also known as Mb21d1) is an enzyme that produces cyclic guanosine monophosphate–adenosine monophosphate (cGMP-AMP or cGAMP), a second messenger that is activated upon binding of cGAS to DNA or RNA:DNA hybrids in the cytoplasm (22, 23). cGAS can induce signaling that is known to promote the up-regulation of inflammatory genes and play a critical role in age-related macular degeneration and cellular senescence (24–26). cGAS-induced cGAMP binds to the endoplasmic reticulum (ER)-associated transmembrane protein STING (which is also known as TMEM173). STING recruits TANK-binding kinase 1 (TBK1), which phosphorylates transcription factors, such as IFN regulatory factor 3 (IRF3) and IFN regulatory factor 7 (IRF7), and other substrates, such as IκB kinase α (IKKα), cRel, and p62 (sequestosome) (27, 28). cGAS also plays a major role in the regulation of autophagy; this indicates that there is a close molecular and signaling link between inflammatory response and autophagy (20, 29–31). The role of cGAS in cancer, diabetes, and immune disorders is well established, but its role in neurodegenerative disease remains less clear (32–34). Up-regulation of cGAMP/STING signaling, however, is linked to dopaminergic and cerebellar neuron degeneration in parkin-deficient and ataxia telangiectasia mice model, respectively (35, 36). But the role of cGAS in HD remains unknown. Using ribosome profiling, biochemical, and molecular biology tools, we investigate the role of cGAS in HD.

## Results

### High Ribosome Occupancy in Exon 1 of the *cGAS* mRNA in HD Cells.

**Fig. 1.**
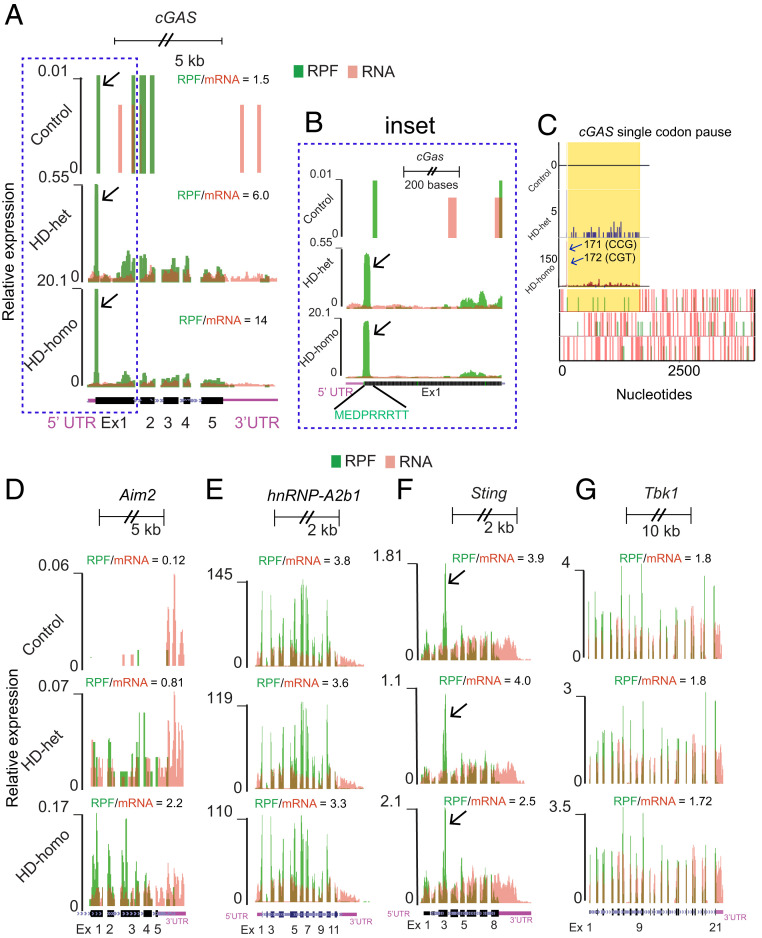
Ribosome occupancy on *cGAS* mRNA transcript in control, HD-het, and HD-homo cells. (*A*) Graphs showing overlay of RPF/mRNA reads for *cGAS* transcript obtained from the UCSC browser. Arrows indicate the position of ribosome occupancy. (*B*) *Inset* showing of exon (Ex) 1 of *cGAS* transcript. Arrows indicate the position of ribosome occupancy. (*C*) Graphs representing codon pause of the cGAS transcripts predicted from PausePred software. Arrows indicate the position of paused codons. (*D*–*G*) Graphs showing overlay of RPF/mRNA reads for *Aim2* (*D*), *hnRNP-A2b1* (*E*), *Sting* (*F*), and *Tbk1* (*G*) *mRNA* transcripts obtained from the UCSC browser. Arrows indicate the expected pause at exon 3 of *Sting* mRNA due to signal peptide insertion into the ER. Ribosome footprints are shown from pooling all three replicates for control, HD-het, and HD-homo cells.

Our study applies high-resolution ribosome sequencing (Ribo-seq) technology for the analysis of genetically precise knockin HD cell models: Immortalized ST*Hdh*^*Q7/Q7*^ (control), ST*Hdh*^*Q7/Q111*^ (HD-het), and ST*Hdh*^*Q111/Q111*^ (HD-homo) striatal neuronal cells derived from WT, *Hdh*^*Q7/Q111*^, and *Hdh*^*Q111/Q111*^ mouse embryos, respectively (37). In our previous study (38), we successfully generated high-quality reads and identified new roles for HTT as a physiological suppressor of translation via regulation of ribosome movement. The global ribosome profiling data of the present study revealed that *cGAS*, which has five exons, showed high ribosome occupancy (based on the ribosome-protected fragment [RPF]/mRNA ratio). The RPF counts in exon 1 of HD-het and HD-homo cells were higher than the counts in control cells (Fig. 1*A*, arrows). The ribosomes accumulated at the 5ʹ end of exon 1 in the region (TAC CTT CTA GGC GCA TCT TCC TGC TGC) that codes for MEDPRRRTT (Fig. 1 *B*, *Inset*, arrows); this indicates that the *cGAS* mRNA is translationally regulated in HD. With the help of a pause prediction software (39), we found an additional single codon pause at 171 (CCG) and 172 (CGT) in the HD cells (Fig. 1*C*, arrows). The gene, absence of melanoma 2 (*Aim2*), another cytosolic DNA sensor (40), was also up-regulated in HD cells, but to a much smaller extent than observed for *cGAS* (the RPF/mRNA for *cGAS* was 14, whereas it was 2.2 for *Aim2* in HD-homo cells) (Fig. 1*D*). Similarly, *hnRNP-A2b1*, a newly identified sensor for viral double-stranded DNA—but not for endogenous DNA—in the nucleus (40), was found to be expressed at a similar level in the control and HD cells (Fig. 1*E*). Other known DNA sensors, such as Toll-like receptor 9, is mostly restricted to blood and immune cells (41) but not expressed in striatal cells (38). We also examined whether the ribosome profile of two major *cGAS* downstream targets, namely *Sting* and *Tbk1*, is altered in HD cells. However, with the exception of a slight reduction in the RPF/mRNA ratio of *Sting* in HD-het cells, we found no major difference in the ribosome occupancy of *Sting* or *Tbk1* between the control and HD cells (Fig. 1 *F* and *G*). Interestingly, *Sting* showed an expected pause at exon 3 (Fig. 1*F*, arrow), which might be necessary in cotranslational translocation for proper insertion into the ER (42, 43). Together, the data indicate that the *cGAS* mRNA is selectively up-regulated in HD cells and shows high ribosome occupancy at exon 1, which may imply potential dysregulation of translation.

### Up-Regulation of the cGAS Pathway in HD.

**Fig. 2.**
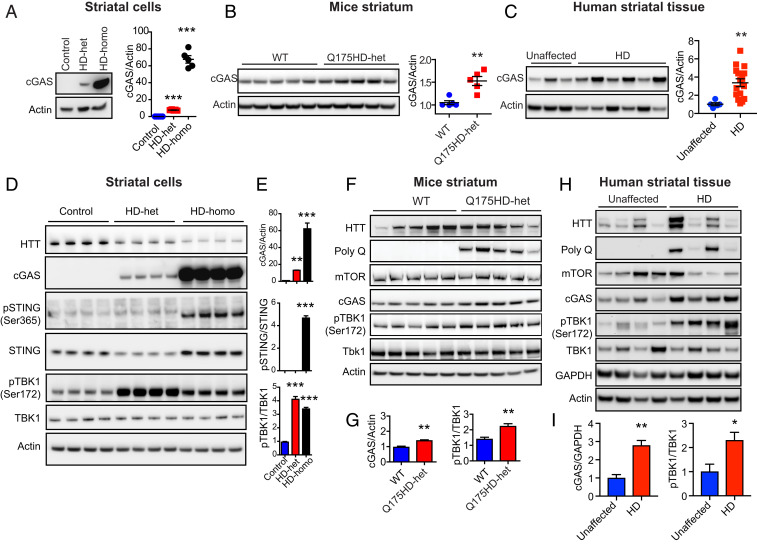
cGAS and its targets genes are up-regulated in HD. (*A*–*C*) Representative Western blots for indicated proteins in the control, HD-het, and HD-homo striatal neuronal cells (*n* = 5, independent experiments) (*A*), WT and Q175HD-het (neo-) striatum (*n* = 5 mice per group) (*B*), human unaffected (*n* = 5) and HD affected (*n* = 16) striatum (*C*). Bar graph represents quantification of indicated proteins, normalized with actin. (*D*) Representative Western blot for indicated proteins in the striatal neuronal cells. (*E*) Bar graph represents quantification of indicated proteins from *D*, normalized with actin. Data presented as mean ± SEM (*n* = 13 control, *n* = 4 HD-het, *n* = 13 HD-homo). (*F*) Representative Western blot for indicated proteins in WT and Q175HD het (neo-) striatum. (*G*) Bar graph represents quantification of indicated proteins from *F*, normalized with actin. Data presented as mean ± SEM (*n* = 5 mice per group). (*H*) Western blots for indicated proteins from human unaffected (control, *n* = 5) and HD affected (*n* = 16) striatum. (*I*) Bar graph represents quantification of indicated proteins from *H*, normalized with GAPDH. Data in the bar graphs represented as mean ± SEM. *P < 0.05, **P < 0.01, ***P < 0.001 by Student’s *t* test (*A*–*C* and *G*) or one-way ANOVA followed by Tukey’s multiple comparison test (*E*).

The high-ribosome occupancy of the *cGAS* mRNA indicates that ribosomes are potentially stalled on exon 1 (Fig. 1 *A*–*C*); this may result in an increase or decrease in protein production. Therefore, our next step was to investigate the protein levels of cGAS in HD cells. Western blot analysis showed that the cGAS protein is robustly up-regulated in HD-het and HD-homo striatal cells, but it is barely found in control striatal cells (Fig. 2*A*). Furthermore, the expression of cGAS in HD-homo cells was 60-fold higher than that in HD-het cells (Fig. 2*A*). cGAS up-regulation was also observed in a Q175HD-het (neo-) striatum (44) (Fig. 2*B*) and in the striatum from human postmortem HD patients (Fig. 2*C*). The data indicate that the cGAS protein is up-regulated in HD; therefore, we investigated its activity.

Following activation of cGAS, cGAMP stimulates STING at the ER, which then promotes translocation of TBK1 to the endosomal and lysosomal compartment and leads to activation of the inflammatory response (45, 46). STING is then is phosphorylated at Ser-365 (pSTING Ser365) by Ulk1, and it then facilitates the phosphorylation of TBK1 at Ser-172 (pTBK1 Ser172) (45, 47, 48). Thus, pSTING (Ser365) and pTBK1 (Ser172) were used as downstream markers for the assessment of activation of cGAS signaling in HD. We observed that the phosphorylation level of STING (Ser365) was high in the HD-homo cells, but pSTING was not detected in control or HD-het cells (Fig. 2 *D* and *E*). Furthermore, phosphorylation of TBK1 (Ser172) was higher in both types of HD striatal cells than in the control cells (Fig. 2 *D* and *E*). We also observed an increase in the pTBK1 (Ser172) levels in Q175HD-het mice, as compared to the WT mice (Fig. 2 *F* and *G*), and in the striatum from HD patients as compared to the striatum from healthy controls (Fig. 2 *H* and *I*). These findings indicate that the expression and activity of cGAS are up-regulated in HD.

### Up-Regulation of cGAS-Dependent Inflammatory Genes in HD.

**Fig. 3.**
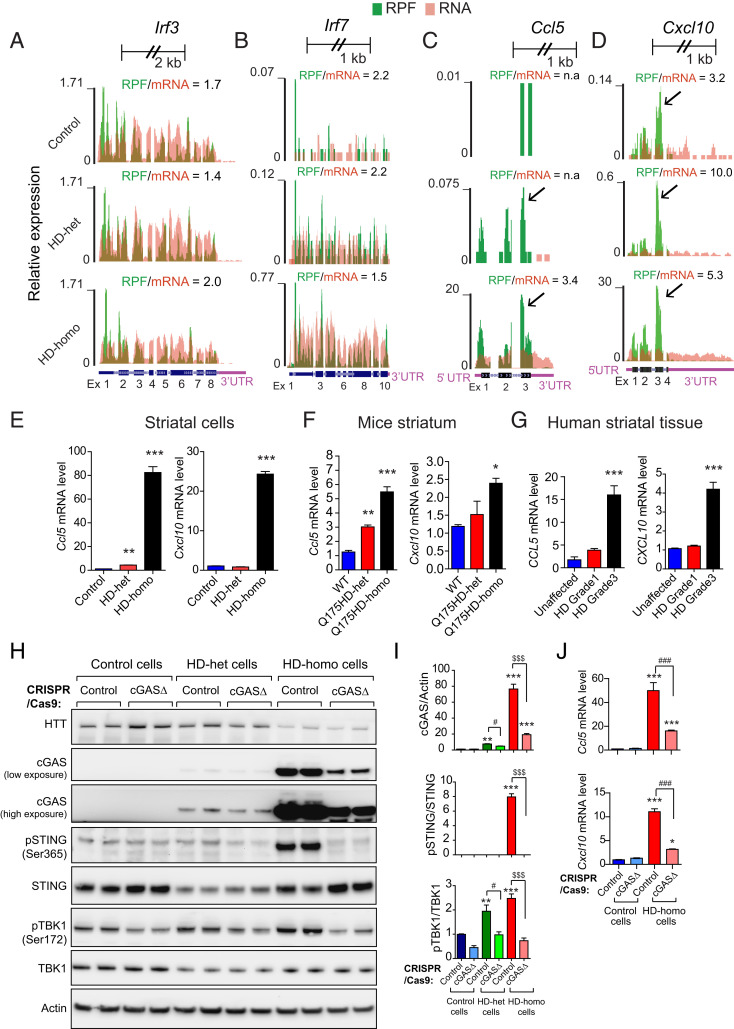
Depletion of cGAS inhibits the inflammatory response in HD cells. (*A*–*D*) Graphs showing overlay of RPF/mRNA reads for *Irf3* (*A*), *Irf7* (*B*), *Ccl5* (*C*)**,** and *Cxcl10* (*D*) *mRNA* transcripts obtained from the UCSC browser. Ribosome footprints are shown from pooling all three replicates for control, HD-het, and HD-homo cells. (*E*–*G*) qPCR analysis of indicated mRNA in striatal cells (*E*), Q175HD (neo-) mouse striatum (*F*), and human striatum (*G*). Bar graph represents quantification of indicated mRNA, normalized with *Gapdh*. Data presented as mean ± SEM, (*n* = 3, independent experiments), *P < 0.05, **P < 0.01, ***P < 0.001 by one-way ANOVA followed by Tukey’s multiple comparison test. (*H*) Representative Western blot of indicated proteins from control, HD-het, and HD-homo striatal neuronal cells that are generated using control CRISPR/CAS-9 (control) or cGAS gRNA expressing CRISPR/CAS-9 cells (cGAS-depleted cells, cGASΔ). (*I*) Bar graph represents quantification of indicated proteins from *H*, normalized with actin. Data presented as mean ± SEM (*n* = 4 to 8 independent experiments), **P < 0.01, ***P < 0.001 compared to control CRISPR/Cas9 in control cells, ^#^*P* < 0.05 between HD-het cells control CRISPR and HD-het cells cGASΔ, and ^$$$^*P* < 0.001 between HD-homo cells control CRISPR and HD-homo cells cGASΔ by one-way ANOVA followed by Tukey’s post hoc test. (*J*) qPCR analysis of *Ccl5* and *Cxcl10* mRNA. Bar graph represents quantification of indicated mRNA, normalized with *Gapdh*. Data presented as mean ± SEM, (*n* = 3, independent experiments), *P < 0.05, ***P < 0.001 compared to contol CRISPR/Cas9 in control cells and ^###^*P* < 0.001, between HD-homo cells control CRISPR and HD-homo cells cGASΔ by one-way ANOVA followed by Tukey’s post hoc test.

Next, we investigated whether the levels of certain known cGAS-regulated inflammatory response genes (49, 50) are affected in HD, with the help of our ribosome profiling data (38). We examined the ribosome profiles of the mRNAs of cGAS-regulated inflammatory transcription factors (*Irf3*, *Irf7*) and inflammatory chemokines (*Ccl5* and *Cxcl10*) in the HD and control cells (Fig. 3 *A*–*D*). We found no major differences in the RPF/mRNA ratio of *Irf3* between HD-homo, HD-het, and control cells, but the RPF/mRNA ratio of *Irf7* was slightly lower in HD-homo cells than in the control cells (1.5 vs. 2.2). This finding indicates that the ribosome occupancy of *Irf7* is slightly decreased, but its mRNA levels are enhanced in HD-homo cells. Thus, there is no major difference in the RPF/mRNA ratio of the two known cGAS-regulated inflammatory transcription factors Irf3 and Irf7 (47, 51) between control and HD cells (Fig. 3 *A* and *B*). In contrast, we found that the RPF/mRNA ratios of *Ccl5* and *Cxcl10* are markedly higher in the HD cells: For *Ccl5*, the RPF/mRNA ratio was 3.5 in the HD-homo cells, while there were none or few reads in the HD-het and control cells. For *Cxcl10*, the RPF/mRNA ratio was 5.3 and 10.0 for the HD-homo and HD-het cells, respectively, and it was 3.2 in the control cells. Thus, enhanced mRNA expression and high ribosome occupancy were observed in the mRNA of the inflammatory chemokines, *Ccl5* and *Cxcl10*, in HD cells. Interestingly, high ribosome occupancy was found on exon 3 of *Ccl5* and *Cxcl10*; this is indicative of translational control of these mRNAs (Fig. 3 *C* and *D*, arrows).

Using qPCR analysis, we confirmed that the *Ccl5* and *Cxcl10* mRNAs are up-regulated in HD-het and HD-homo striatal cells (Fig. 3*E*) and in the Q175HD-het and Q175HD-homo mouse striatum (Fig. 3*F*), as well as striatum of grade 3 human HD patients (Fig. 3*G*). Although qPCR analysis showed that the *Ccl5* and *Cxcl10* mRNAs are not robustly altered in HD-het striatal cells (Fig. 3 *E* and *F*), we cannot exclude the possibility that the *Ccl5* or *Cxcl10* protein levels are increased in HD-het striatal cells, compared to the controls, because they show enhanced ribosome occupancy (Fig. 3 *C* and *D*). Collectively, these data indicate mRNA up-regulation of cGAS-dependent inflammatory response genes and altered ribosome occupancy of their mRNA in HD.

### Effect of Depletion of cGAS on pSTING and pTBK1 Levels and Inflammatory Response in HD Cells.

To further establish a causal role for cGAS in the inflammatory response in HD, we explored whether depletion of cGAS could revert the inflammatory response. To this end, we transfected CRISPR/Cas9 gRNA directed against cGAS (cGAS depletion or cGASΔ) in the control, HD-het, and HD-homo striatal cells. We used nonspecific CRISPR/Cas9 gRNA (control gRNA) for the control groups. The cGASΔ HD-homo cells resulted in more than 80% depletion of cGAS (Fig. 3 *H* and *I*), as well as almost complete elimination of pSTING (Ser365) (Fig. 3 *H* and *I*) and significant reduction of pTBK1 (Ser172), which was also decreased in cGASΔ-HD-het cells (Fig. 3 *H* and *I*). Notably, pSTING (Ser365) was observed at negligible levels in the control and HD-het cells, presumably due to low cGAS levels (Fig. 3 *H* and *J*). Together, these findings indicate that cGAS depletion reduces the activation of its downstream targets, pSTING and pTBK1, in HD cells.

Next, we investigated whether cGAS depletion also interferes with the up-regulation of the inflammatory response in HD. For this, we performed qPCR analysis of *Ccl5* and *Cxcl10* in cGASΔ-HD-homo and control-HD-homo cells. cGAS depletion dramatically attenuated *Ccl5* (Fig. 3*I*) and *Cxcl10* (Fig. 3*J*) expression in the cGASΔ-HD-homo cells compared to the control-HD-homo cells. Taken together, these findings indicate that cGAS up-regulation is causally linked to the activation of its downstream signals as well as the inflammatory response.

### Differences in LC3A and LC3B Levels and Ribosome Occupancy in HD Cells.

Previous studies indicate that autophagy is dysregulated in HD, and it is known that HTT plays several roles in regulating the dynamics of autophagy (21, 52). During autophagy in mice, Atg proteases are cleaved at the glycine site (G) of pro-LC3A at ETF**G**F(stop) and pro-LC3B at ETF**G**TAMAV(stop) to generate LC3A-I and LC3B-I, respectively (53, 54). LC3A-I and LC3B-I are further lipidated by the Atg enzyme to produce LC3A-II and LC3B-II, which bind to autophagosomes and initiate autophagy (55).

**Fig. 4.**
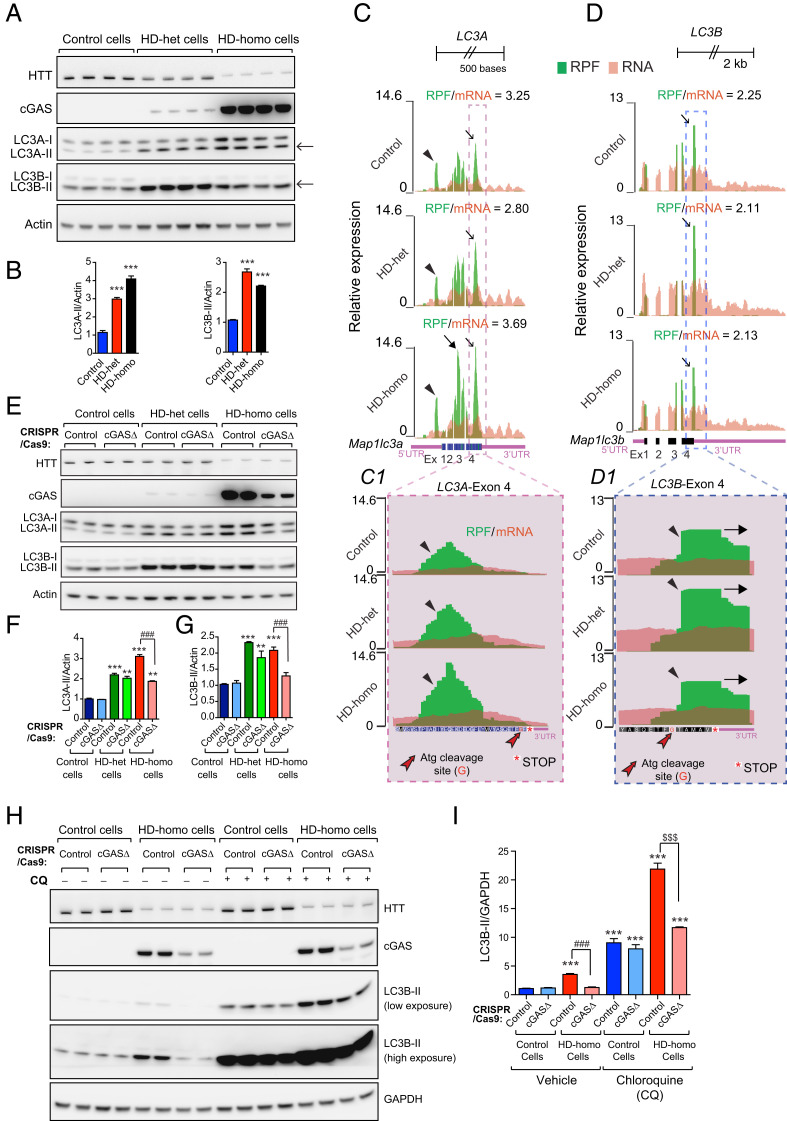
cGAS regulates autophagy in HD cells. (*A*) Representative Western blot of indicated proteins from control, HD-het, and HD-homo striatal neuronal cells. Arrows indicate LC3A-II and LC3B-II. (*B*) Bar graph represents quantification of indicated proteins from *A*, normalized with actin. Data presented as mean ± SEM, (*n* = 4, independent experiments), ***P < 0.001 compared to control cells by one-way ANOVA followed by Tukey’s post hoc test. (*C* and *D*) Graphs showing overlay of RPF/mRNA reads for *LC3-A* (*C*) and *LC3B* (*D*) transcript obtained from the UCSC browser. *Insets C1* and *D1* show exon 4 of *LC3-A* and *LC3-B* transcript. Arrowheads indicate the position of ribosome occupancy and red arrow shows Atg cleavage site (Glycine, G). Ribosome footprints are shown from pooling all three replicates for control, HD-het, and HD-homo cells. (*E*) Representative Western blot of indicated proteins from control, HD-het, and HD-homo striatal neuronal cells that are generated using control CRISPR/CAS-9 (control) or cGAS gRNA expressing CRISPR/CAS-9 cells (cGAS-depleted cells, cGASΔ). (*F* and *G*) Bar graph represents quantification of LC3A-II (*F*) and LC3B-II (*G*) proteins from *E*, normalized with actin. Data presented as mean ± SEM (*n* = 3 independent experiments), **P < 0.01, ***P < 0.001 compared to control CRISPR/Cas9 in control cells, ^###^*P* < 0.001 between control CRISPR and cGASΔ in HD-homo cells by by one-way ANOVA test followed by Tukey’s post hoc test. (*H*) Western blots of indicated proteins from control cells and HD-homo cells treated with vehicle or chloroquine (CQ, 4 h, 50 μM). (*I*) Bar graph represents quantification LC3B-II from *H*, normalized with GAPDH. Data presented as mean ± SEM (*n* = 3, independent experiments), ***P < 0.001 compared to control CRISPR/Cas9 in control cells, ^###^*P* < 0.001 between contol CRISPR (vehicle) and cGASΔ (vehicle) in HD-homo cells and ^$$$^*P* < 0.001 between control CRISPR and cGASΔ in HD cells after CQ treatment, by one-way ANOVA followed by Tukey’s multiple comparison.

First, we used Western blot analysis to examine the status of LC3A and LC3B conversion in HD and control striatal cells, with the help of antibodies that detects both the I and II forms of LC3A and LC3B. In general, we observed higher basal LC3B-II levels than LC3A-II levels in all of the groups of striatal cells (Fig. 4*A*, arrows). However, both the LC3A-II and LC3B-II levels were higher in the HD cells than in the control cells (Fig. 4 *A* and *B*). This finding indicates that the steady-state basal autophagy levels are higher in HD cells than in control cells, as reported in previous studies (56–58).

Next, we explored why the basal LC3B-II conversion was higher than the basal LC3A-II conversion in HD cells (Fig. 4*A*, arrows). We speculated whether this difference was attributable to differences in the proforms of LC3A and LC3B in HD cells. As there are no antibodies available for the detection of the mouse or human proforms of LC3A and LC3B, we used the RPF/mRNA data (an indicator of ribosome occupancy) for *LC3A* and *LC3B* in the HD and control striatal cells (38) as an indirect measurement of the levels of the uncleaved proforms of LC3A and LC3B. As shown in Fig. 4*C*, the *LC3A* mRNA, which has four exons, showed high ribosome occupancy in the 5ʹUTR (Fig. 4*C*, arrowheads), and in exon 3 and exon 4 (Fig. 4*C*, thin arrows) (compared to exons 1 and 2). The ribosome occupancy was slightly higher in the HD-homo cells (Fig. 4*C*, closed arrow) than in the HD-het and control cells. Moreover, the ribosomes appear stalled on exon 4 (Fig. 4 *C*, *C1 Inset*, arrowheads) just before the Atg cleavage site (ETF**G**F) and the stop codon (Fig. 4 *C*, *C1 Inset*), both in the HD and control cells. Next, the RPF/mRNA profile of *LC3B* exhibited four exons. High ribosome occupancy was observed on exon 4 (compared to exons 1, 2, and 3) in both the HD and control cells (Fig. 4*D*, arrowheads). Interestingly, in contrast to LC3A, the 5ʹUTR of LC3B did not show ribosome occupancy (Fig. 4*D*). Furthermore, in contrast to LC3A, the ribosomes appear stalled on exon 4 after the cleavage site of the protease Atg (Fig. 4 *D*, *D1 Inset*, arrowheads), and appeared to read through the stop codon and further into the 3ʹUTR (Fig. 4 *D*, *D1 Inset*, arrow).

Taken together, the RPF/mRNA data of LC3A and LC3B indicate that they are translationally regulated by the regulation of the ribosome occupancy, which might explain the higher basal levels of LC3B-II than LC3A-II in HD cells.

### Role of cGAS in Mediating Autophagy Flux in HD Cells.

Previous studies indicate that the cGAS/STING pathway also regulates autophagy (29–31), which is a major catabolic process affected in HD (21, 59). We, therefore, investigated the role of cGAS in the regulation of autophagy in HD cells. To this end, we examined basal autophagy in cGASΔ-HD-het, cGASΔ-HD-homo, and cGASΔ-control cells. The cGASΔ-HD-homo cells showed a robust reduction in LC3A-II (Fig. 4 *E* and *F*) and LC3B-II (Fig. 4 *E* and *G*), and the cGASΔ-HD-het cells showed a slight decrease in LC3A-II (Fig. 4 *E* and *F*) and LC3B-II (Fig. 4 *E* and *G*). This result indicates that cGAS up-regulation controls the autophagy response in HD cells.

Next, we investigated whether cGAS modulates autophagy flux in HD, as previous studies suggest that HD cells show enhanced autophagy flux as well as cargo-loading defects (56, 58, 60). We treated cGASΔ-HD-homo cells and control cells with chloroquine (CQ), which is a known inhibitor of autophagy flux (61, 62). As shown in Fig. 4 *H* and *I*, we observed an ∼2.5-fold increase in LC3B-II conversion in HD-homo cells compared to control cells, and this difference was further enhanced in the presence of CQ. This finding indicates that basal autophagy flux is increased in HD. Finally, cGAS depletion (cGASΔ) in both vehicle- and CQ-treated HD-homo cells resulted in a decrease in LC3B-II formation compared to the corresponding control cells (Fig. 4 *H* and *I*). Together these results indicate that cGAS promotes autophagy flux in HD by enhancing LC3B-II formation (i.e., the formation of autophagosomes).

### Effect of Reconstitution of cGAS on the Autophagy and Inflammatory Response in cGAS-Depleted HD Cells.

**Fig. 5.**
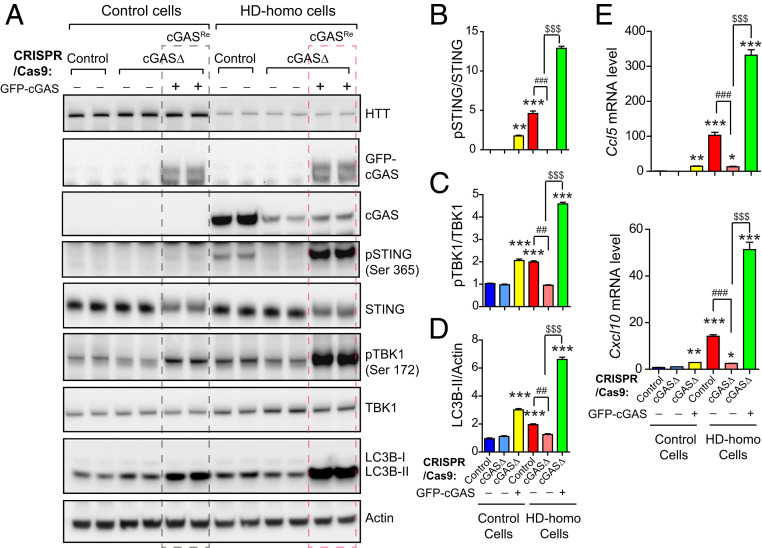
cGAS reconstitution in cGAS-depleted HD striatal neuronal cells reactivates cGAS signaling and inflammatory responses. (*A*) Representative Western blot of indicated proteins from control and HD-homo striatal neuronal cells that are generated using control CRISPR/CAS-9 (control) or cGAS gRNA expressing CRISPR/CAS-9 cells (cGAS-depleted cells, cGASΔ). cGASΔ control cells or HD-homo cells were transfected with GFP-cGAS as indicated. (*B*–*D*) Bar graph represents quantification of indicated proteins from *A*, normalized with actin. Data presented as mean ± SEM, (*n* = 3, independent experiments), **P < 0.01, ***P < 0.001 compared to control CRISPR/Cas9 in control cells, ^##^*P* < 0.01, ^###^*P* < 0.001 between control CRISPR/Cas9 and cGASΔ in HD-homo cells and ^$$$^*P* < 0.001 between HD-homo cells cGASΔ and HD-homo cells cGASΔ with GFP-cGAS reconstitution by one-way ANOVA followed by Tukey’s post hoc test. (*E*) qPCR analysis of *Ccl5* and *Cxcl10* mRNA. Bar graph represents quantification of indicated mRNA, normalized with *Gapdh*. Data presented as mean ± SEM, (*n* = 3, independent experiments), *P < 0.05, **P < 0.01, ***P < 0.001 compared to control CRISPR/Cas9 in control cells, ^###^*P* < 0.001 between control CRISPR/Cas9 and cGASΔ in HD-homo cells and ^$$$^*P* < 0.001 between HD-homo cells cGASΔ and HD-homo cells cGASΔ with GFP-cGAS reconstitution by one-way ANOVA followed by Tukey’s post hoc test.

Next, we introduced exogenous GFP-tagged cGAS (GFP-cGAS) into cGASΔ-HD-homo cells and cGASΔ-control cells to confirm the role of cGAS in autophagy and inflammatory responses in HD. After the reconstitution of cGAS (cGAS^Re^) in the cGASΔ-HD-homo cells, we observed robust induction of pSTING (Ser365) (Fig. 5 *A* and *B*) and pTBK1 (Ser172) (Fig. 5 *A* and *C*). In addition, cGAS^Re^ induced robust up-regulation of autophagy, as indicated by the LC3B-II conversion, in the cGASΔ-HD-homo cells (Fig. 5 *A* and *D*). cGAS^Re^ in the cGASΔ-HD-homo cells also resulted in an increase in the mRNA levels of the inflammatory response genes *Ccl5* and *Cxcl10*, compared to the control cells (Fig. 5*E*). Notably, cGAS^Re^ in the cGASΔ-control cells also induced cGAS activity, and the autophagy and inflammatory responses, albeit at a lower level compared to HD-homo cells (Fig. 5). This finding indicates that mHTT is required for enhanced cGAS activity.

Together, these results indicate that cGAS reconstitution restores cGAS activity, and autophagy and inflammatory responses in cGAS-depleted HD cells. Thus, cGAS may play a critical role in the pathogenesis of HD.

### cGAS Enrichment in Lysosomal and Mitochondrial Fractions and Coimmunoprecipitation with mHTT.

**Fig. 6.**
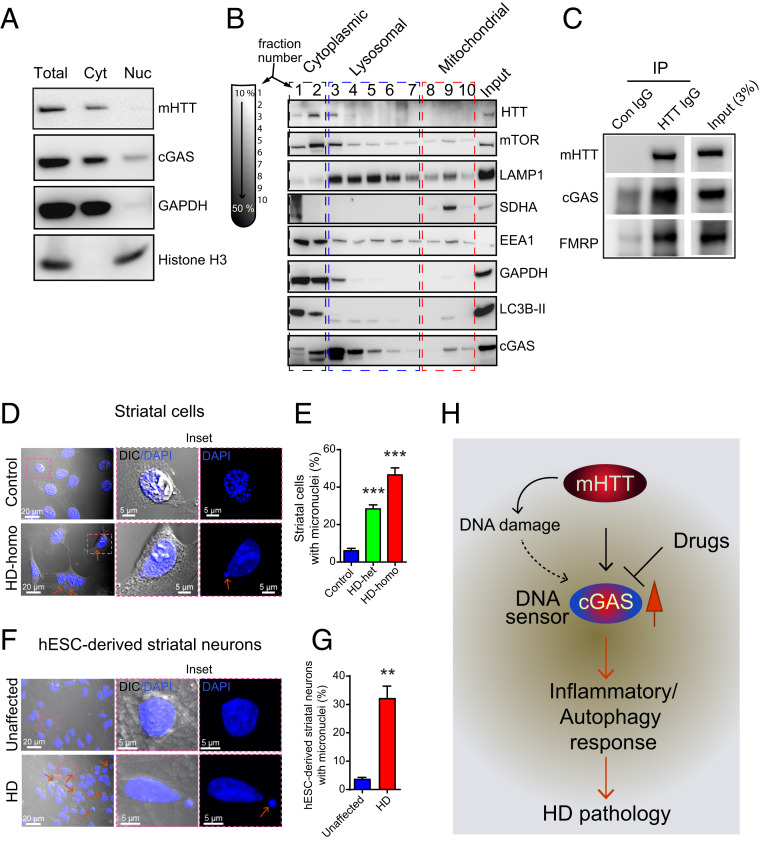
HD cells harbor cytoplasmic micronuclei. (*A*) Representative Western blot of indicated proteins, lysed from cytoplasmic (C) and nuclear (N) fractions from HD-homo striatal neuronal cells. Total is whole-cell lysate. (*B*) Western blots for indicated proteins isolated from the fractions obtained from sucrose density based gradient centrifugation of HD-homo cells. GAPDH and LAMP1 antibody was used to label cytoplasmic and lysosomal fractions, respectively. Mitochondrial fraction was labeled by SDHA antibody. (*C*) Western blot showing mHTT and cGAS binding in HD-homo cells. Cell lysate was immunoprecipitated by either control IgG antibody or HTT antibody and probed for HTT, cGAS, and FMRP. Blot is representative of three independent experiments. (*D*) Representative confocal and differential interference contrast (DIC) images of striatal cells acquired using Zeiss LSM 880 microscope. *Inset* is magnified area from selected region. Arrows represent micronuclei in cytoplasm. (*E*) Bar graph represents quantification of micronuclei in control, HD-het, and HD-homo cells from *D*. Data presented as mean ± SEM (control = 308 cells, HD-het = 458 cells, HD-homo = 134 cells), ***P < 0.001 by one-way ANOVA followed by Tukey’s post hoc test. (*F*) Representative confocal and DIC images of hESC-derived striatal neurons. *Inset* is magnified area from selected region. Arrow indicates micronuclei in the cytoplasm. (*G*) Bar graph represents quantification of micronuclei in unaffected and HD hESC-derived neurons from *F*. Data represented as mean ± SEM (unaffected = 569 neurons, HD = 403 neurons) **P < 0.01 by Student’s *t* test. (*H*) Model depicts mHTT expressing cells increases DNA sensor cGAS levels that controls inflammatory and autophagy responses and thus HD pathogenesis.

Previous studies have reported that cGAS is localized to the nucleus, plasma membrane, or intercellular organelles (63–65). We investigated the localization of cGAS in HD striatal cells by using a biochemical method (Fig. 6*A*). We found that cGAS was enriched in the cytoplasm, and a considerable amount was also detected in the nuclear fractions (Fig. 6*A*). To further investigate the intracellular localization of cGAS in the cytoplasm, we performed density-based gradient centrifugation and subcellular separation of organelles (Fig. 6*B*). We found that the cGAS-containing fractions sedimented at lighter density fractions that were enriched in autophagic/lysosomal markers and LC3B-II/LAMP1, as well as, to some extent in higher density fractions that were enriched in mitochondrial marker, SDHA (Fig. 6*B*). Thus, cGAS is enriched in the cytoplasm, lysosomal, and mitochondrial fractions in HD-homo striatal cells.

As mHTT interacts with multiple proteins, we examined whether mHTT also interacts with cGAS. Immunoprecipitation assay showed that cGAS does coimmunoprecipitate with mHTT. FMRP, a protein that is known to interact with mHTT, was also detected in the coimmunoprecipitated mHTT (Fig. 6*C*) (38). This finding indicates that mHTT interacts with cGAS and may regulate its activity.

### Enhanced Cytoplasmic Micronuclei in HD.

Finally, we considered the question of how cGAS is up-regulated. A previous study showed that cGAS can be up-regulated in the presence of cytoplasmic micronuclei (42), which are discrete DNA fragments that are independent of the main nucleus and may be indicative of DNA damage (66). Since we found rapid up-regulation of cGAS levels and activity in HD cells, we explored the possibility of micronuclei being present in HD cells. Indeed, we found numerous micronuclei in the HD-homo cells (43% of the cells), HD-het cells (30% of the cells) compared to the control cells (5% of the cells) (Fig. 6 *D* and *E* and *SI Appendix*, Fig. S1). Similarly, human HD striatal neurons, derived from human embryonic stem cells (hESCs), also showed numerous cytoplasmic micronuclei compared to neurons derived from unaffected hESCs (Fig. 6 *F* and *G*). We identified these neurons as medium spiny neurons (MSNs) as they were positive for DARPP-32, which is a marker of MSN (*SI Appendix*, Fig. S2). Together, these data indicate that HD cells harbor numerous micronuclei, which might contribute to the up-regulation of cGAS and its activity in HD.

## Discussion

The data presented here demonstrate that cGAS is up-regulated in HD and is causally linked to the promotion of inflammatory and autophagy responses that might contribute to HD pathology (Fig. 6*H*). Our working model predict that the DNA damage/repair observed in HD (67–70) may be a starting point for cGAS up-regulation (Fig. 6*H*). However, the mechanisms of DNA damage and repair or the formation of micronuclei, as discovered in this report and in an earlier report in HD (71), and how they trigger cGAS induction remain less clear (42, 46, 67).

Intriguingly, our Ribo-Seq and biochemical data showed that the ribosome occupancy of *cGAS* mRNA and its protein levels and activity are more enhanced in homozygous HD cells (HD-homo) than in heterozygous HD (HD-het) cells (Figs. 1 and 2). Thus, cGAS induction may be dependent on the copy number of mHTT. However, the mechanisms that contribute to the high ribosome occupancy in exon 1 of cGAS are unclear. Recently, we demonstrated that mHTT promotes stalling of ribosomes and inhibits global protein synthesis (38), and at the same time, the translation of select mRNA, such as *Fmr1* mRNA, is increased in HD. Therefore, it is possible that mHTT directly regulates the translation of *cGAS* mRNA via the modulation of ribosome occupancy on exon 1. Alternatively, cGAS mRNA may be transcriptionally induced in response to DNA damage and repair (67–70). In fact, this may enhance exon 1 ribosome occupancy and cGAS production. However, it is not clear whether high ribosome occupancy on exon 1 of cGAS is restricted to HD cells or whether it is a ubiquitous “ribosome signature” of *cGAS* mRNA in all cell types.

With regard to the mechanism by which cGAS promotes inflammatory response in HD, it is known that the active cGAS-STING pathway activates TBK1, which phosphorylates the transcription factors IRF3/IRF7, and these factors, in turn, regulate the inflammatory cytokines Ccl5 and Cxcl10 (47, 72, 73). The active TBK1 can also promote Ccl5 and Cxcl10 expression via Tlr signaling; thus, the inflammatory responses in HD may involve cGAS-dependent and cGAS-independent pathways. Previous studies have reported that Ccl5 and Cxcl10 are up-regulated in various HD models (74, 75). Interestingly, mHTT has been shown to inhibit the secretion of the Ccl5 protein, as immunocytochemical analysis showed stronger intracellular Ccl5 protein accumulation in HD astrocytes than in WT astrocytes (76). Our data indicate high ribosome occupancy on exon 3 of *Ccl5* in HD cells (Fig. 3*C*). The high ribosome occupancy of *Ccl5* could contribute to its defective secretion in HD, but this needs to be explored in a future study. Importantly, mHTT in astrocytes has been shown to influence the pathogenesis of HD, and physiological cGAS expression has been found to be higher in astrocytes than in neurons (77–79). Thus, cGAS might orchestrate the inflammatory response in HD via the canonical STING/TBK1 signaling pathway in nonneuronal cells.

Our study findings demonstrate the critical role of cGAS in the regulation of autophagy in HD cells. Previously, it was demonstrated that cGAS induced the activation and translocation of STING to the ER–Golgi intermediate compartment, which serves as the source for LC3-II formation (29). Interestingly, HTT can act as a scaffold for autophagy initiation by bringing together other autophagy regulators, such as p62 and ULK1 kinase, and promoting the formation of autophagosomes (80–82). Autophagy flux has been shown to be increased in HD (57, 58, 83), but HD cells also demonstrate defects in cargo delivery and loading into autophagosomes (84). Thus, the mechanisms of autophagy flux or its defects in HD are not fully understood (21, 59). We found high basal levels of LC3A-II and LC3B-II in HD striatal cells, compared to control cells, and demonstrated that HD cells show enhanced autophagy flux (Fig. 4). As cGAS is a known inducer of autophagy flux via enhancement of LC3-II formation (29, 30), we propose that cGAS may serve as a major regulator of autophagy flux in HD. However, it is not clear whether the cGAS-STING pathway alone is responsible for the autophagy flux in HD, or whether cGAS and mHTT affect different domains of the autophagy flux.

Although TBK1 has been shown to play a major role in cGAS-mediated inflammatory response, it has been demonstrated that cGAS-STING–mediated autophagy flux is TBK1-independent (29). However, TBK1 is involved in promoting autophagosome maturation (85). In this report, we demonstrated that TBK1 is highly up-regulated in HD cells, and its activity is dependent on cGAS. However, the HD-het cells displayed TBK1 activation that was high and comparable to that of HD-homo cells, even though they had very low cGAS levels (Fig. 2 *A* and *D*). Based on these findings, we propose that aberrant autophagy in HD can occur in a cGAS-dependent or TBK1-dependent fashion, and can affect both autophagosome formation and maturation. Moreover, mHTT, like TBK1, also regulates p62, NF-kB signaling, and mechanistic target of rapamycin complex 1 (mTORC1) signaling (27, 28, 86–92). Thus, mHTT and TBK1 may play a synergistic role in HD progression via more than one pathway.

Based on our Ribo-seq data, which is derived from isolating actively translating 80S ribosomes (38), for *LC3A* and *LC3B* mRNA, we predict the following: the ribosome occupancy on the 5ʹUTR of *LC3A* (Fig. 4*C*) may indicate that LC3A is translated through the 5ʹUTR or that it is involved in 5ʹUTR-dependent translational regulation (93–96). Furthermore, the ribosomes that accumulate after the cleavage site of the conserved protease Atg on exon 4 of *LC3B* mRNA and stop-codon read-through (Fig. 4*D*) may serve as a site for pro-LC3B cleavage by Atg to generate LC3B-I (97). Such a cotranslational mechanism may occur, for example, by the recruitment of protease Atg to the stop-codon read-through stalled ribosomes to generate the LC3B-I needed for lipidation and autophagy initiation. Analogous cotranslational proteolytic events are described for N-terminal methionine excision events that unmask glycine for lipidation, which is required for cell-signaling mechanisms (98). Therefore, we predict that LC3A and LC3B may be differentially regulated via cotranslational proteolytic events to initiate autophagy, which might additionally contribute to the cGAS-mediated autophagy in HD.

The striatum is the most vulnerable region in HD. However, the mechanisms that promotes striatal damage remain unclear, as do the mechanisms via which cGAS activation in HD regulates striatal vulnerability. We speculate that in the striatum, Rhes, a GTPase/SUMOE3-like protein, which increases mHTT solubility and promotes cell-to-cell transport of mHTT via tunneling-nanotube–like membrane protrusions, might participate in cGAS activity and propagate inflammatory and autophagy responses via intercellular signaling (99–104).

Our mechanistic model, which is based on data integrated from ribosome profiling, and molecular and biochemical approaches, provides deep insight into the causal role of cGAS signaling in fostering inflammatory and autophagic responses in the HD cell model. Thus, our study illustrates the translational potential of cGAS inhibition in alleviating HD symptoms and progression.

## Materials and Methods

### Cell Culture, Antibodies, Plasmids, and Human Tissue.

Mouse ST*Hdh*^*Q7/Q7*^ (control), ST*Hdh*^*Q7/Q111*^ (HD-het), and ST*Hdh*^*Q111/Q111*^ (HD-homo) striatal neuronal cells (37) were obtained from the Coriell Institute and cultured as described in our previous works (92, 101, 105). For autophagy flux measurements, 50 μM CQ (dissolved in water) was added to cells for 4 h. Details about antibodies plasmids and human tissue used in the study can be found in *SI Appendix*. cDNA preparation, real-time PCR, nuclear/cytoplasmic and subcellular fractionation, Western blotting, and imaging details can be found in *SI Appendix*.

### hESC-Derived Neuron Culture.

Unaffected (Genea-019) and HD-affected (HTT-48Q, Genea-020) hESC lines were obtained from the Cure Huntington Disease Initiative. The lines were grown and expanded in feeder-free conditions on vitronectin using Essential 8 plus medium (Thermo Fisher Scientific) at 37 °C and 5% CO_2_. Medium was replaced daily. hESCs were differentiated into striatal neurons using a previously described protocol (106) (*SI Appendix*).

### Ribosome Profiling.

RNase foot printing, generation of cDNA libraries from ribosome protected mRNAs, generation of cDNA libraries and sequencing, generation of mRNA-seq libraries, Ribo-seq, RNA-seq quality control, and mapping the reads to the University of California, Santa Cruz (UCSC) browser and ribosome pause analysis, were carried out as described in our previous work (38). A full UCSC Genome Browser link for the global ribosome footprints pooling all three replicates for control, HD-het and HD-homo cells can be found at https://genome.ucsc.edu/cgi-bin/hgTracks?hubUrl=Https://de.cyverse.org/anon-files/iplant/home/rmi2lab/Hub_Collaborations/Srini/hub.txt&genome=mm10.

### Mice.

C57BL/6J (WT) and the zQ175 neo-deleted knockin mouse (B6J.129S1-Htt^tm1.1Mfc^/190ChdiJ, stock no. 029928) obtained from Jackson Laboratories and maintained in our animal facility according to Institutional Animal Care and Use Committee at The Scripps Research Institute. Three- to 4-mo-old mice were used for the experiment.

### Generation of cGAS-Depleted Striatal Cells.

cGAS-depleted striatal cells (cGASΔ) were generated using cGAS CRISPR/Cas9 plasmids from Santa Cruz Biotechnologies, as described previously (99, 100). (*SI Appendix*).

### Statistical Analysis.

Unless otherwise noted, all experiments were carried out in duplicates and repeated at least three times. The statistical comparison was carried out between groups using one-way ANOVA followed by Tukey’s multiple comparison test or Student’s *t* test, and significance values were set at *P* < 0.05, using GraphPad Prism 7.

### Data Availability.

Sequencing data have been submitted to the Gene Expression Omnibus (GEO) data repository, under accession no. GSE146674.

## Supplementary Material

Supplementary File
