# Supplementary material for: Cyclic GMP-AMP synthase promotes the inflammatory and autophagy responses in Huntington disease
Source: Proc Natl Acad Sci U S A. 2020 Jun 24;117(27):15989–99. doi: 10.1073/pnas.2002144117 (PMC7354937; doi:10.1073/pnas.2002144117)
Supplement: Supplementary File [file pnas.2002144117.sapp.pdf]

SI Appendix

SI Methods

Antibodies, Plasmids and treatment

Huntingtin (MAB2166) and PolyQ (P1874) antibodies were obtained from Millipore-Sigma. Actin (sc-47778), GAPDH (sc-365062), cGAS (sc-515777) and DARPP-32 (sc-271111) antibodies were purchased from Santa Cruz Biotechnology. Huntingtin (5656), mTOR (2983), FMRP (4317), LC3A (4599), LC3B (3868), STING (50494), phospho-STING Ser365 (72971), cGAS (31659), TBK1 (3504), phospho-TBK1 Ser172 (5483), IRF3 (4302), phospho-IRF3 Ser396 (29047), LAMP1 (9091), SDHA (11998), EEA1 (2411), and MAP2 (8707) antibodies were purchased from Cell Signaling Technology. HRP-conjugated secondary antibodies [goat anti-mouse (115-035-146) or goat anti-rabbit (111-035-144)] were from Jackson ImmunoResearch Inc. Alexa Fluor 488 and Alex Fluor 568-conjugated secondary antibodies for immunocytochemistry were from Thermo Fisher Scientific. pTRIP-SFFV-EGFP-FLAG-cGAS was a gift from Nicolas Manel (Addgene plasmid #127661) PSL Research University, INSERM, Paris, France.

hESC-derived neuron culture

hESCs were differentiated into striatal neurons using a previously described protocol (SI ref. 1). Briefly, on Day 0, hESCs were detached with Versene to form embryoid bodies (EBs) in Neural induction medium (NIM, DMEM/F12: Neurobasal plus (1:1), 1x N2 supplement, 1x B27 supplement, 1x GlutaMAX, 1x penicillin streptomycin) plus LDN-193189 (100 nM, Sigma), SB431542 (10 μM, Sigma), XAV939 (4 μM, Selleck), SAG (100 nM, Selleck). The EBs were then plated in neural proliferation medium (NPM, DMEM/F12: Neurobasal plus (1:1), 0.5x N2 supplement, 1x B27 supplement, 1x GlutaMAX, 1x penicillin streptomycin) plus LDN-193189 (100 nM), SB431542 (10 μM), XAV939 (4 μM), SAG (100 nM) onto plastic plates coated with polyornithine (15 μg/ml, Sigma)/laminin (5 μg/ml, Sigma)/fibronectin (5 μg/ml, Thermo fisher Scientific) on Day 4. Next day the medium was changed to NPM medium plus LDN-193189 (100 nM), SB431542 (10 μM). Medium was changed every other day. On Day 9, medium was changed to NPM medium without any small molecules. Within days 11–16 of differentiation, the cells were dissociated with Accutase and replated onto polyornithine/fibronectin/laminin-coated

coverslips in neural differentiation medium (NDM, Neurobasal plus, 1x B27 supplement, 1x GlutaMAX, 1x penicillin streptomycin) plus ascorbic acid (AA, 0.2 mM, Sigma), and DAPT (1μM, Tocris), BDNF (20 ng/ml, Prospec), GDNF(10 ng/ml, Prospec). The medium was changed every other day with NDM medium supplemented with AA (0.2 mM), and DAPT (1μM), BDNF (20 ng/ml), GDNF (10 ng/ml). The differentiated striatal neurons on Day 22 were used for staining with DAPI and immunostaining with MAP2, DARPP32 antibodies as described in our earlier studies (SI ref. 2, 3).

Human HD patient samples

Human brain tissue (Caudate nucleus) samples of grade 1 HD-affected patient (HSB # 3358, 2706, 3744), grade 2 HD-affected patient (HSB # 2858, 3432, 3635, 3872, 4072, 3159), grade 3 HD-affected patient (HSB # 4344, 2869, 2972, 4518, 4254), grade 4 HD-affected patient (HSB # 5078, 2903) and normal donor controls (HSB # 4615, 4823, 5293, 4340, 4135) were obtained from the Human Brain and Spinal Fluid Resource Center VA West Los Angeles Healthcare Center, Los Angeles, CA.

Generation of cGAS depleted striatal cells

First, we transfected the striatal neuronal cells (control, HD-het and HD-homo) with cGAS CRISPR/Cas9 plasmid (sc-437363) or CRISPR/Cas9 control plasmid (sc-418922) in 10cm dish. After 48 h we sorted the cells based on GFP fluorescence and re-cultured them. We passaged them 2-3 time and prepared lysate to confirm the cGAS depletion by Western blotting using cGAS antibody. Exogenous pTRIP-SFFV-EGFP-FLAG-cGAS (GFP-cGAS) was transfected into the cGASΔ-HD-homo cells and cGASΔ-control cells to further investigate the role of cGAS in autophagy and inflammatory responses in HD.

Western blotting

To check the protein expression level striatal neuronal cells (control and HD) were plated in 6 well plate ($2x10^5$ cells per well). After 24 hours, cells were washed in PBS and lysed in buffer containing 1% Triton X-100 in 50 mM Tris-HCl, pH 7.4, 150 mM NaCl and 1x protease inhibitor cocktail (Roche, Sigma) and 1x phosphatase inhibitor (PhosStop,

Roche, Sigma), sonicated for 3 x 5 sec at 20% amplitude, and cleared by centrifugation for 10 min at 11,000g at 4°C . Protein concentration was determined with a bicinchoninic acid (BCA) protein assay reagent (Pierce). Equal amounts of protein (30-40 μg) were loaded and were separated by electrophoresis in 4 to 12% Bis-Tris Gel (Thermo Fisher Scientific), transferred to polyvinylidene difluoride membranes, and probed with the indicated antibodies. HRP-conjugated secondary antibodies (Jackson ImmunoResearch Inc.) were probed to detect bound primary IgG with a chemiluminescence imager (Alpha Innotech) using enhanced chemiluminescence from WesternBright Quantum (Advansta) (SI ref. 4). The band intensities were quantified with ImageJ software (NIH). Phosphorylated proteins were then normalized against the total protein levels (normalized to actin or GAPDH). Mice striatal tissues were homogenized in radioimmunoprecipitation assay (RIPA) buffer [50 mM Tris-HCl (pH 7.4), 150 mM NaCl, 1.0% Triton X-100, 0.5% sodium deoxycholate, 0.1% SDS,] with 1x complete protease inhibitor cocktail (Roche, Sigma) and 1x phosphatase inhibitor (PhosStop, Roche, Sigma), followed by a brief sonication for 2 x 5 sec at 20% amplitude and cleared by centrifugation for 10 min at 11,000g at 4°C. Protein estimation was done using a BCA method and proceeded to Western Blotting as mentioned above. Immunoprecipitation from HD-homo cells was carried out using HTT antibody as described before (SI ref. 5). Human tissue was homogenized in lysis buffer [50 mM tris (pH 7.4), 150 mM NaCl, 10% glycerol, and 1.0% Triton X-100] with protease and phosphatase inhibitors, followed by a brief sonication for 6 s at 20% amplitude and cleared by centrifugation for 10 min at 11,000g at 4°C. Protein estimation was done using a BCA method and proceeded to Western Blotting as mentioned above.

Real-time PCR

RNA was extracted from the fractionated samples following lysis in Trizol reagent. 250 ng RNA was used to prepare cDNA using Takara PrimeScript 1st strand cDNA Synthesis Kit (Cat no. 6110A) using random hexamers. The qRT-PCR of genes was performed with SYBR green (Takara RR420A) reagents. Primers for all the genes were designed based on sequences available from the Harvard qPCR primer bank. The primer sequences are as follows:

Gapdh mouse (Forward primer) 5' primer AGGTCGGTGTGAACGGATTTG
(Reverse primer) 3' primer TGTAGACCATGTAGTTGAGGTCA
Ccl5 mouse (Forward primer) 5' primer GCTGCTTTGCCTACCTCTCC
(Reverse primer) 3' primer TCGAGTGACAAACACGACTGC
Cxcl10 mouse (Forward primer) 5' primer CCAAGTGCTGCCGTCATTTTC
(Reverse primer) 3' primer GGCTCGCAGGGATGATTTCAA
GAPDH human (Forward primer) 5' primer GGAGCGAGATCCCTCCAAAAT
(Reverse primer) 3' primer GGCTGTTGTCATACTTCTCATGG
CCL5 human (Forward primer) 5' primer CCAGCAGTCGTCTTTGTCAC
(Reverse primer) 3' primer CTCTGGGTTGGCACACACTT
CXCL10 human (Forward primer) 5' primer CCAATTTTGTCCACGTGTTG
(Reverse primer) 3' primer TTCTTGATGGCCTTCGATTC

Nuclear/cytoplasmic and Subcellular Fractionation

For subcellular fractionation, HD-homo cells were trypsinized and lysed in buffer A of mitochondria isolation buffer (Thermo Fisher Scientific 89874) and kept on ice for 2 minutes. Buffer C was added to each sample and mixed by inverting 5 times. The homogenates were centrifuged at low speed (700g) for 10 minutes to separate nuclei and cell debris. The supernatants were immediately loaded on top of 10-50% sucrose gradients and centrifuged at 40000 RPM (SW41Ti rotor) at 4°C for 2 hours. The gradients were fractionated manually (10 x 1 ml fractions). Using methanol/chloroform, total protein of each fraction was precipitated. The protein pellets were resuspended in 2x LDS/β-mercaptoethanol lysis buffer and proceeded to Western Blotting as mentioned above.

Staining with DAPI

Striatal neuronal cells (Control and HD-het, HD-homo) plated in glass bottom dishes or hESC-derived striatal neurons on coverslips were washed in D-PBS and fixed for 10 min in 4% PFA (Electron Microscopy Sciences). The cells were washed 3 times with D-PBS and incubated with DAPI (4′,6-diamidino-2-phenylindole, Sigma) for 10 min.

Image processing and micronuclei quantification

For control and HD-homo cells, fluorescent confocal images were taken in Zeiss LSM 880 microscope using 63X oil immersion Plan- apochromat objective (1.4 NA). Excitation was via a 405 nm diode-pumped solid-state laser. Pinholes were set so that the section thickness was equal for all channels and ≤ 1 AU. Images were acquired with an optimal Z-step of 0.27 μm covering the whole cellular volume. Processing was performed with Zen software black/blue edition 2012. HD-het cells were imaged using Leica DM5500B microscope (100x objective). For micronuclei quantification, we counted the cells with small DAPI positive puncta separated from nucleus.

SI References

SI ref. 1 Wu M*, et al.* (2018) A Chemical Recipe for Generation of Clinical-Grade Striatal Neurons from hESCs. *Stem Cell Reports* 11(3):635-650.

SI ref. 2 Shahani N, Subramaniam S, Wolf T, Tackenberg C, & Brandt R (2006) Tau aggregation and progressive neuronal degeneration in the absence of changes in spine density and morphology after targeted expression of Alzheimer's disease-relevant tau constructs in organotypic hippocampal slices. *J Neurosci* 26(22):6103-6114.

SI ref. 3 Pryor WM*, et al.* (2014) Huntingtin promotes mTORC1 signaling in the pathogenesis of Huntington's disease. *Sci Signal* 7(349):ra103.

SI ref. 4 Shahani N*, et al.* (2016) RasGRP1 promotes amphetamine-induced motor behavior through a Rhes interaction network ("Rhesactome") in the striatum. *Sci Signal* 9(454):ra111.

SI ref. 5 Eshraghi M*, et al.* (2020) RasGRP1 is a causal factor in the development of l-DOPA-induced dyskinesia in Parkinson's disease. *Sci Adv* 6(18):eaaz7001.

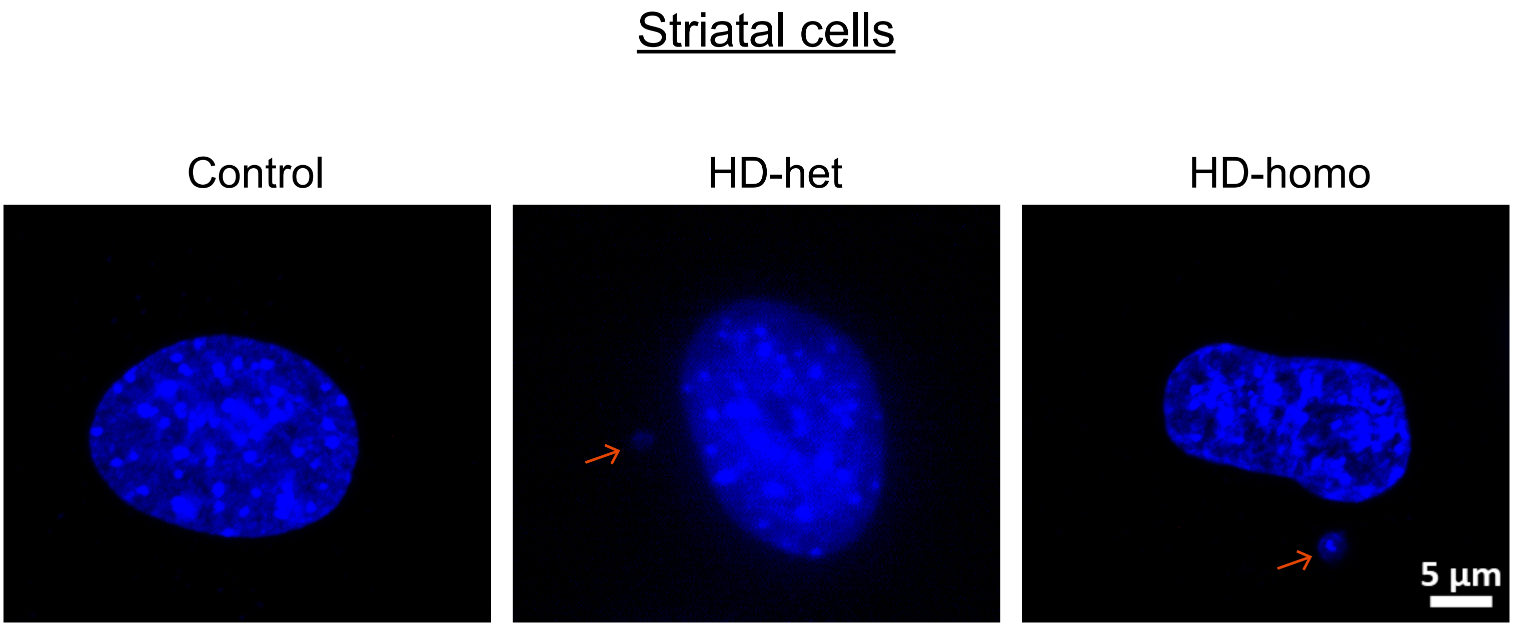

Fig. S1. Representative fluorescent image (for HD-het cell) acquired using Leica DM5500B microscope (100x objective) or of control and HD-homo cells (acquired using Zeiss LSM 880 microscope) stained with DAPI. Arrow shows presence of micronuclei in cytoplasm.

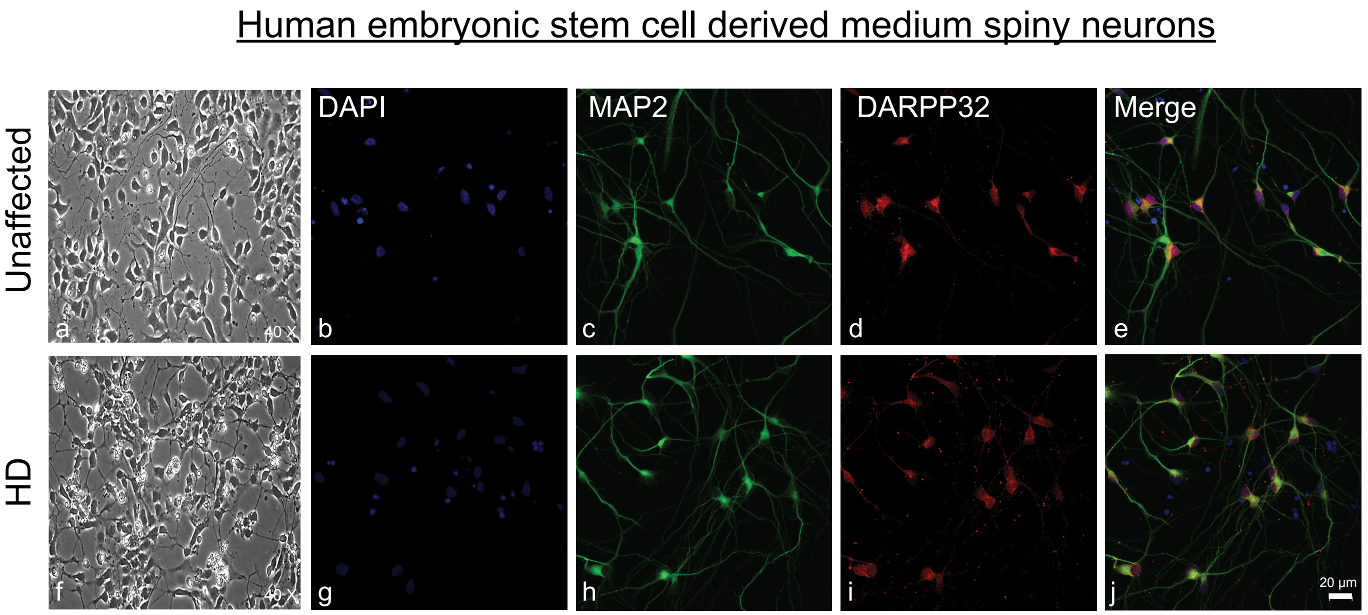

Fig. S2. **Embryonic Stem Cells Derived Medium Spiny Neurons**: A- (a & f) Phase contrast photomicrograph of medium spiny neurons derived from Healthy ESC (unaffected) and HD (48 CAG repeats) ESC at Day 25 of differentiation. (b-e, g-j) Confocal photomicrographs showing MAP2 and DARPP32 immunostaining confirming the identity of medium spiny neurons derived from Healthy and HD ESC acquired using Zeiss LSM 880 microscope.
